# Factors Influencing Energy Drink Usage Amongst Pupils in the Mahikeng Sub-District, Northwest

**DOI:** 10.3390/nu17050770

**Published:** 2025-02-21

**Authors:** Karabo Dina Thini, Kebogile Elizabeth Mokwena, Mohora Feida Malebatja

**Affiliations:** Department of Public Health, School of Healthcare Sciences, Sefako Makgatho Health Sciences University, Pretoria Ga-Rankuwa 0208, South Africa; 202302034@swave.smu.ac.za (K.D.T.); kebogile.mokwena@smu.ac.za (K.E.M.)

**Keywords:** energy drinks usage, caffeine, addictions, substance abuse, pupils, adolescents, Mahikeng sub-district

## Abstract

**Background/Objectives:** The high consumption rate of energy drinks among pupils is a serious public health concern in various countries, including South Africa. Excessive consumption of energy drinks that contain elevated caffeine and sugar levels has the potential to lead to the development of addictions, strokes, dehydration, sleeping disorders, mental health and central nervous disorders, hypertension, digestive problems, and anxiety. Most pupils regard energy drinks as regular soft drinks and lack knowledge of the active ingredients contained in energy drinks and their side effects. The objective of this study was to investigate factors influencing energy drink usage amongst pupils in the Mahikeng sub-district, Northwest Province. **Methods**: A quantitative cross-sectional survey was conducted amongst 505 pupils in the Mahikeng sub-district, Northwest, using self-administered questionnaires. Data were analysed using STATA software version 18 to examine associations between variables. **Results**: The energy drinks consumed most by pupils were Dragon (38.21%), Switch (28.97%), and Red Bull (14.62%). Factors and reasons influencing energy drink usage among pupils include all-night parties (3.1%), concentration (20.3%), being awake (43.1%), curiosity (2.2%), energy levels (23.1%), exams (13.8%), sports (8.7%), fatigue (6.9%), and health (2.3%). There was a strong association (*p* ≤ 0.05) between energy drink usage and sports activities amongst pupils. **Conclusions**: It is concluded that health education and promotion intervention programmes are required to educate pupils about the dangers of energy drink usage to prevent public health risks. Further studies, including research on primary school pupils, are necessary, considering that a substantial number of pupils were exposed to energy drinks at an early age.

## 1. Introduction

The use of energy drinks among learners is a serious public health concern in both developed and developing countries, including South Africa [1,2,3,4]. Energy drinks are highly caffeinated drinks with elevated levels of sugars and other derivatives such as vitamins, amino acids, and herbal extracts [5,6]. The key ingredient in these beverages is caffeine, which can vary considerably according to different brands and possibly exceed the recommended daily caffeine intake [7]. Energy drinks entered the beverage markets with catchy names, hitting the shelves in Europe around the 1980s and quickly spreading worldwide when Red Bull was introduced in the 1990s [8]. Their advertising tactics often involve catchy brand names and attractive messages to their target market, particularly young consumers, offering them a wide range of exciting benefits [9,10].

Various studies that have investigated the prevalence of energy drink consumption among school learners revealed varying rates across different regions and age groups of learners [11,12]. It is known that the secondary school era is a crucial stage in the lives of learners, marked by a rapid growth phase that involves physical growth, maturation, and physiological changes such as brain development [13,14,15]. At this stage and age, learners are also known to be in a risk-taking period, developing new habits of which consuming energy drinks has been one of the predominant habits among them [16,17,18].

Although energy drinks are promoted as performance enhancers and lifestyle beverages, they pose risks in diverse demographics [16,17,18]. The surge in the use of energy drinks, particularly among young people, has raised concerns about their potential adverse health effects and the broader implications for public health systems [19].

The country was reported to have a combined prevalence of overweight and obesity in children aged 6–14 years; this was reported to be 13.5%, which is higher than the 10% global prevalence in school children [20].

Energy drinks as a sugar-sweetened beverage contain high levels of caffeine and sugar [5,6]. Excessive consumption of caffeine and sugar such as that found in sugar-sweetened beverages has been associated with obesity and the risk of developing Non-Communicable Diseases NCDs [21].

The prevalence of energy drink usage is relatively high among secondary school learners; this is associated with various negative health outcomes over a prolonged period [16,17,22]. The energy drink consumption rates and frequencies are often triggered by various factors and reasons influencing their overall health in a manner that is not beneficial [23,24,25].

Studies revealed that most secondary school learners and energy drink consumers in general use these drinks for reasons such as enhancing their sporting performance, meeting the demands of academic life, and upholding social lifestyle standards [2,26,27,28]. Studies have reported that 6.19% and 11.7% of learners, respectively, are using energy drinks for academic performance [26,29]. Furthermore, another study found that 7.79% of the learners consume these drinks before school and 12.06% consume them during school hours [30].

The caffeine content in energy drinks has been reported to stimulate the central nervous system, leading to increased cognitive functioning, such as alertness, improved memory, and speed reaction time [31]. This can be particularly advantageous for long study periods to assist in delaying fatigue and is often an attraction for learners and students.

Another common reason for the usage of energy drinks includes involvement in sporting activities such as athletics to improve their performance [28,32,33,34]. Various studies published on the use of energy drinks particularly by learners participating in sports reported that between 20% and 70% of athletes consume these beverages [35,36,37,38]. There is a strong consensus that energy drinks can positively impact sports performance. Studies have shown positive outcomes on the relationship between physical performance in sports and the consumption of energy drinks [6,39,40].

In Africa, it would seem that the reasons may differ slightly compared to the European and Asian regions as it appears that energy drinks are primarily consumed for refreshment purposes rather than for energy and performance effects [2].

Taste appears to be the most reported social reason for the consumption of energy drinks, as studies report that the primary reason for some learners to consume energy drinks is their taste [26,29]. Furthermore, authors have reported that fewer learners consumed them due to peer pressure, to imitate celebrities, and for courtesy of others and politeness [23,36,41]. Other scholars have reported that the consumption of energy drinks amongst consumers is often triggered by nothing, meaning there is absolutely no particular reason or reason related to this practice [22,42], and that it may just be a habit.

Factors such as exams, sports, peer pressure, curiosity, keeping awake, energy boost, concentration levels, and fatigue were reported to be some of the factors influencing and contributing to the high consumption rates and usage of energy drinks amongst pupils in various locations globally [16,33,34,41,43]. A gap has been identified that there is limited literature published on energy drink usage, particularly amongst pupils in South Africa. There is limited knowledge pertaining to energy drink usage health effects amongst consumers particularly learners [18,36,44]. Therefore, this study aimed to investigate factors influencing energy drink usage amongst pupils in the Mahikeng sub-district, Northwest.

## 2. Materials and Methods

### 2.1. The Study Area

**Figure 1:**
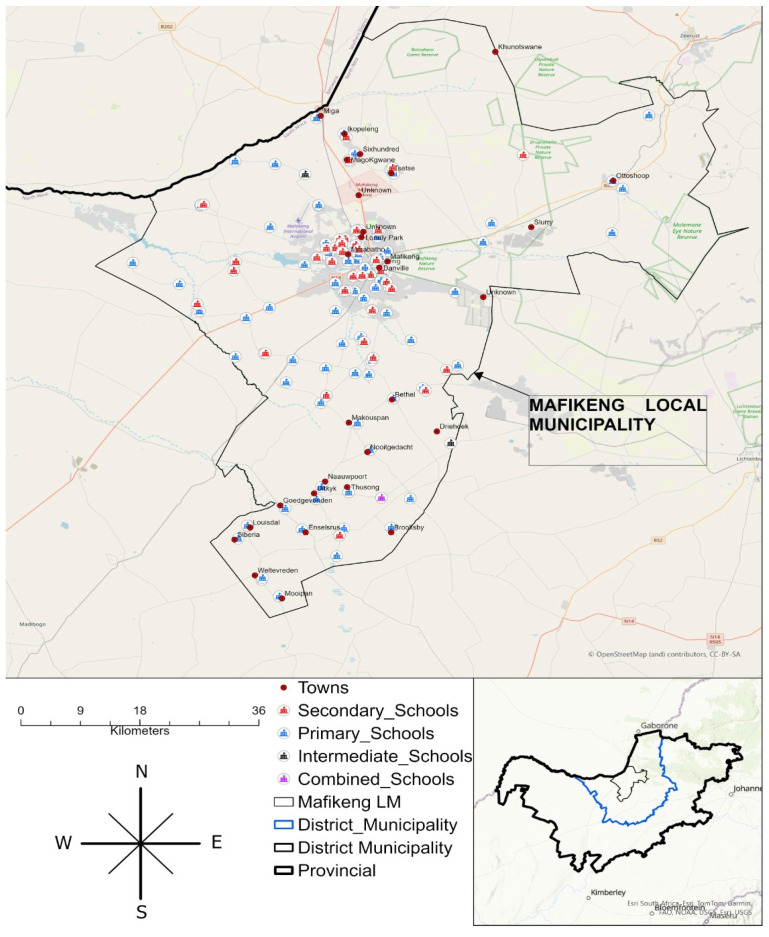
Map of the Mahikeng Sub-District.

The study was conducted in secondary schools in the Mahikeng sub-district. The Mahikeng sub-district falls under Ngaka Modiri Molema District (NMMD) (see Figure 1), which has a population estimation of about 314,394 [45]. The Mahikeng sub-district comprises 150 schools, with 136 public schools, 14 private or independent schools [46], and 48 secondary schools enrolling learners in grades 8 to 12. The common languages spoken in the Mahikeng sub-district include Setswana, English, and Afrikaans [45]. The Mahikeng sub-district is dominated by a majority of Africans, followed by a minority of Mixed race, Whites, and Asians. Secondary schools located in areas such as Lotlhakane, Majemantsho, Magogoe, Seweding, Motlhabeng, Montshioa, Danville, Golf View, Lonely Park, and Lokaleng were targeted as the study locations under the Mahikeng sub-district which have extensive learners that consume energy drinks.

### 2.2. Study Design, Population, and Sample Selection

A quantitative cross-sectional survey was conducted in schools in the Mahikeng sub-district, Northwest Province. The study design utilized primary data collected from secondary school learners to investigate the prevalence of usage of energy drinks among school learners. This approach allowed the researcher to collect data at one point in time, making it efficient, cost-effective, and less time-consuming [47].

**Inclusion Criteria**

The study included male and female learners ranging from the ages of 12 years to the eldest learners in schools who are enrolled from grade 8 to grade 12 in secondary schools in the Mahikeng sub-district of Northwest Province and whose parents consented to participation.

**Exclusion Criteria**

The study excluded male and female learners attending secondary schools in the Mahikeng sub-district of Northwest Province who fit the criteria but were not willing to participate. All learners enrolled in secondary schools in the Mahikeng sub-district of Northwest Province who fit the criteria but have medical conditions limiting or contraindicating the usage of energy drinks were excluded from the study.

The researcher scheduled an appointment with school Principals and Heads of departments to formally request permission to conduct the study in the schools located in the Mahikeng sub-district. Information leaflets (Supplementary S1) and Informed consent forms (Supplementary S2) were disseminated to the parents by the schools to request parents of secondary school learners to give consent to participate in the study. Participants were recruited during an assembly at each sampled school on the dates agreed by the school management. The purpose of the study, significance, aim, objectives, and information leaflets were discussed in detail with the potential participants during recruitment. Participants who agreed to form part of the study, and who fit the criteria were requested to sign written assent forms (Supplementary S3).

### 2.3. Sample Size and Sampling Technique

**Figure 2:**
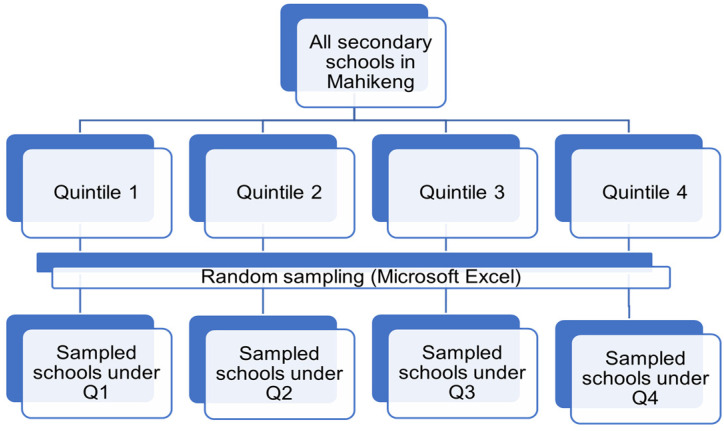
Multi-stage sampling technique applied in the study.

A multi-stage sampling was used to select study participants (refer to Figure 2). The first stage involved the selection of all secondary schools in the Mahikeng sub-district. The next stage grouped the schools according to the quintile ranking under which they are classified. In the third stage, the researcher selected schools from each quintile category by means of simple random sampling, where a random number-generating formula (Microsoft Excel spreadsheet) was used to assist with the random selection of schools for each group. In order to avoid over or under-sampling, the sampling percentage calculation was applied on the basis of the total number of learners per selected school. The researcher reached a sample size of n = 505. The rationale for using a multi-stage sampling approach was to obtain representative samples from large and geographically dispersed populations in the Mahikeng sub-district, Northwest.

### 2.4. Data Collection Tool, Piloting and Procedure

#### 2.4.1. Data Collection Tool

The data were collected using self-administered questionnaires. The questionnaires were written in English and translated into other languages understood by the participants, which are Setswana and Afrikaans. The sociodemographic questionnaire (Supplementary S4) collected the sociodemographic characteristics of the participants. The main data collection questionnaire (Supplementary S5) was adopted from previous similar studies conducted on the consumption patterns and adverse effects of energy drinks and edited by the researcher to address the key objectives of this research [3,48,49]. This questionnaire comprised three sections of 17 closed-ended questions where participants selected their choice using a tick. Where the answers provided did not apply to the participants, an option of “other” which allowed them to specify their response was also provided. The first section aimed to determine the use of energy drinks by identifying the types of energy drinks consumed, how often they were consumed, and how much was consumed by learners. Sections B and C sought to find the reasons related to the usage of energy drinks by the learners and assessed their level of knowledge about energy drinks, respectively.

#### 2.4.2. Piloting

A pilot study was conducted prior to the actual study. The researcher conducted a pilot study at a school in the Mahikeng sub-district that was not sampled. The questionnaire was piloted to ensure that the questions were written in a way that participants understood to verify the feasibility of the questionnaire, to ensure it measured what it was designed for. Twenty participants, contributing to 10% of the minimum sample size completed the questionnaire and their responses were used to test the tool and ensure the validity, practicality, and sensitivity of the tool before the actual data collection. The response rate was 100%, as no participant refused to complete the questionnaire. The participants understood the questions and were able to complete them without difficulty. The researcher adjusted parts of the response options in the questionnaire with the permission of the supervisor after the pilot study. The edited parts were responses about the frequency of energy drink consumption in the last 7 days.

### 2.5. Data Collection

Data collection took 3 months between April and July 2024. Data collection was conducted in the classrooms and halls provided by the school management. In other schools, the researcher moved from room to room to avoid congestion. Each participant took approximately 30 min to 45 to complete their questionnaire.

### 2.6. Data Analysis

Data were analysed using STATA software, version 18. The researcher used a Microsoft Excel spreadsheet to capture and clean data collected from questionnaires. The data were then exported to STATA for statistical tests and analysis. Independent variables in this study were the sociodemographic characteristics, and the uses of energy drinks were the dependent variables. Sociodemographic variables were descriptively analysed to determine the frequencies, means, and variance, which were presented in a table. Univariate analysis was conducted to describe the dependent variables, which were presented in graphs and tables. The usage of energy drinks was categorised according to frequency (e.g., daily, once per week, once per month, etc.), the quantity of consumption which was assessed based on the number of energy drinks consumed in a single setting, and the preferred types of energy drinks were identified by the brands consumed. Bivariate analysis was used to analyse the relation of a dependent variable e.g., frequency of consumption of energy drinks against other independent variables such as age, gender, and grade. Correlation tests were used to assess the association between the current status of energy drink consumption, frequency of consumption of energy drinks, and the reasons for usage of energy drinks against independent variables and other dependent variables. The Pearson chi-square tests of association were conducted to assess the general significance of the association between the variables. The statistical significance was set at *p* ≤ 0.05 and a multivariate analysis was performed on the variables that showed a significant difference to build a logistic regression model.

### 2.7. Reliability, Validity and Bias

The study adhered to standard procedures of data collection procedures to ensure reliability [50]. The researcher received data collection training from the supervisor before commencing with data collection to guarantee the accuracy and reliability of data collection. Pre-testing of the tool was conducted on a school in the Mahikeng sub-district that was not sampled to ensure that the aim and objectives of the study were met. The tool underwent further modifications by the supervisor and researcher before the actual data collection commenced.

### 2.8. Validity

The research questionnaire utilized to conduct the study was adopted from previously validated questionnaires used in similar published studies [3,48,49]. The questionnaire was translated into the language understood by the participants. Internal validity was ensured by involving only participants who met the inclusion criteria. The researcher and the supervisors checked that the questions in the tools were correctly structured to collect the required information to ensure face validity. The data collection tool was assessed for content validity by requesting the assistance of expert personnel in the Department of Public Health experienced in the nutrition and food field.

### 2.9. Bias

Selection bias was minimized by sampling using the survey sampling method, using the minimum sample size, and sampling learners from several schools in different grades within each school. Non-response bias was reduced by translating the questionnaire into the native languages and using simple and clear questions. To avoid information bias, the study’s aim and objectives were clearly explained to the participants, and participants were given clear instructions without guiding their answers to avoid giving false information.

### 2.10. Ethical Considerations

Research clearance approval was obtained from the Sefako Makgatho Health Sciences University Research Ethics Committee (SMUREC/H/22/2024:PG) on 7 February 2024. Permission to conduct the study in the secondary schools of the Mahikeng sub-district was sought from the Department of Basic Education in the Northwest Province, the District Director, and the school management of the sampled schools. Research ethical principles such as privacy, confidentiality, respect, informed consent, protection of personal information, and voluntary participation were observed in this study.

## 3. Results

### 3.1. Profile of the Schools

The total sample of 505 participants came from nine public secondary schools in the Mahikeng sub-district of Northwest Province, covering schools from quintile 1 to quintile 4. The minimum participation rate per school was 22, and the highest was 91. The profile of the schools is presented in Table 1 below.

### 3.2. Socio-Demographics Information of Participants

The socio-demographic characteristics of the participants are presented in Table 2. A total of 505 learners in grades 8 to 12 in nine secondary schools in the Mahikeng sub-district participated in the study. The ages of the learners ranged from 13 to 24 years, with a mean age of 16.86, and a standard deviation of 2.20 years. Almost two-thirds of the participants were females (n = 324, 64.16%). Most of the learners were Black (n = 481, 95.25%), resided in a rural area (n = 417, 82.57%), walked to school (n = 301, 59.60%), and did not participate in any form of sports at school (n = 352, 69.70%). Among those who participated in sports (n = 153, 30.30%), soccer (n = 67, 43.79%), and netball (n = 60, 39.22%) were the most played sports categories. Those who stayed with one parent (n = 206, 40.79%) were more than those who stayed with both parents (n = 175, 34.65%). The rest of the socio-demographic characteristics are reflected in Table 2 below.

### 3.3. Types of Energy Drinks Consumed by Learners

**Figure 3:**
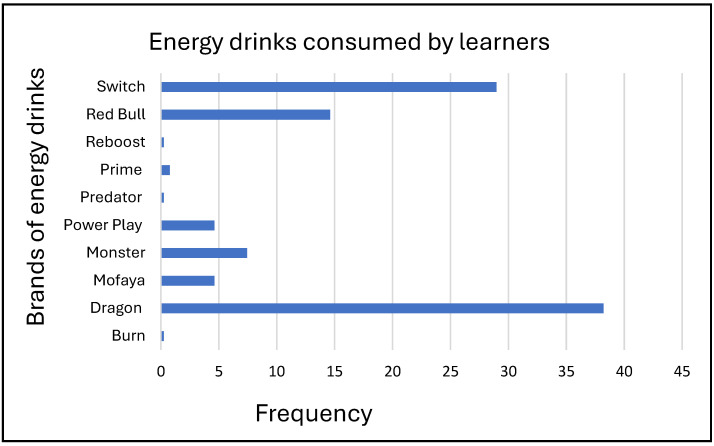
Energy drinks consumed by learners.

The most preferred energy drink brands consumed by learners were Dragon (38.21%), Switch (28.97%), and Red Bull (14.62%) as presented in Figure 3. The most common reason for the choice of a brand was taste (75.38%), as shown in Figure 3 below.

### 3.4. Reasons for Choice of Energy Drink Brand

**Figure 4:**
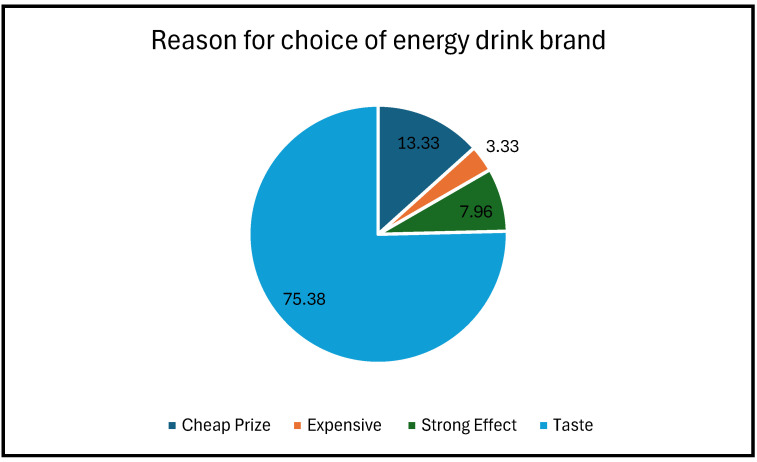
Reasons for choosing an energy drink brand.

Many participants indicated that their choice of energy drink brand is based on its taste, followed by affordability. The minority chose an energy drink brand based on its expensiveness and effect (refer to Figure 4) below.

### 3.5. Reasons for the Use of Energy Drinks

As presented in Table 3, most learners use energy drinks to help them stay awake (43.1%), followed by giving energy (23.1%), and helping them to concentrate during study (20.3%). Less reported reasons for the consumption of energy drinks included out of curiosity (2.1%), to feel healthy (2.3%), and to enjoy an all-night party (3.1%). The majority (n = 224, 57.44) of the learners who currently consume energy drinks reported that their desired effects are sometimes attained after drinking the beverage. Seventy-six (19.49%) learners report that their desired effects are always attained, while 30 (7.69%) of the learners stated that their desired effects are never attained, and 60 (15.38%) do not know whether their desired effects are attained or not after consuming energy drinks.

### 3.6. Factors Associated with the Current Consumption of Energy Drinks

Table 4 shows the factors that were found to be significant when associated with the current consumption status of energy drinks by learners in a bivariate analysis. An association was significantly (*p* ≤ 0.05) established with grade and participation in sports using the Pearson chi-square test of associations. The test further revealed a significant relationship between the knowledge score of the effects of energy drinks and current energy consumption status.

A logistic regression model was built using the three factors identified by the association test. Only two factors remained statistically significant (*p*-value ≤ 0.05) after multivariate analysis, i.e., sports and knowledge score. The results are outlined in (Table 5) below.

### 3.7. Factors Associated with Frequency of Consumption

In response to the research question as to how frequently learners consume energy drinks, the Pearson chi-square test of association identified seven factors that showed a significant relation with the frequency of energy drink consumption (*p* ≤ 0.05), i.e., school, quintile, age category, the quantity of energy drink consumed in a day, effects attainment and reasons for consumption of energy drinks in particular, exam, and no reason (Table 6).

Using the seven identified factors that were significant at Pearson chi-square level analysis, a logistic regression model was built, and only three factors remained statistically significant when associated with the frequency of consumption of energy drinks i.e., age category, the attainment of effects, and exam as the reason for consumption (*p* = 0.05). The results are shown in (Table 7) below.

### 3.8. Factors Associated with the Reasons for the Usage of Energy Drinks

The Pearson chi-square test explored the association between the reasons for consumption related to sociodemographic characteristics and other dependent variables. School and quintile were the two sociodemographic variables significantly associated with the reasons for consumption. Additionally, the tests found a significant association with the attainment of effects, brand, and the quantity of energy drinks in one day. Table 8 below details the association.

A logistic regression analysis was conducted using the related factors to determine which ones would sustain significance, but none of the factors proved to be significant in the model. Further insights are provided in Table 9 below.

## 4. Discussion

Dragon was the main brand, drunk by 38.21% of the respondents, followed by Switch at 28.97% and Red Bull at 14.62%. This pattern of brand preferences among young consumers appears to be consistent with a study on the common energy drinks found in South African retail markets [3]. Red Bull, on the other hand, is expensive compared to other brands, making it less accessible to learners with limited financial resources. Brand preference was primarily determined by taste (75.38%), implying that taste contributes to purchasing behaviour among learners which is consistent with findings in other studies [51]. Price and perceived effect played smaller roles, suggesting that functionality and sensory appeal trump status for this group of consumers.

Most of the participants reported using energy drinks to stay awake, which is similar to the reasons provided in other studies, showing that learners use energy drinks primarily to combat sleepiness and promote vigilance while studying [2,27]. However, the effect of staying awake comes at a price because the secondary caffeine content in energy drinks results in health challenges that include lack of concentration, high blood pressure, and even addiction [17,52]. Even more concerning is that regular use of energy drinks is significantly associated with poor quality of sleep [16,53], which requires the users to increase their consumption of energy drinks, hence creating a vicious cycle. Other rarely reported reasons for consumption, such as drinking out of curiosity, for health reasons, or social activities such as parties, accounted for lower percentages. The results suggest that the reasons for the consumption of energy drinks by the learners are both functional and recreational, aligning with the response to a perceived need for energy, and social or lifestyle purposes [51].

The frequency of use of energy drinks and the amount consumed per day showed a positive association, consistent with previous research indicating that frequent consumption is often associated with an increase in total intake [51]. As a result, the more a learner uses energy drinks in their daily routine, the more likely they are to increase their daily intake. Thus, running the potential risk of negative health outcomes such as insomnia, anxiety, and difficulty concentrating [16,53].

A commonly reported reason for consuming energy drinks was exam preparation, suggesting an association of academic pressures with energy drink consumption. This contrasts with the study by [54] who found that energy drink consumption was rather associated with leisure time. Furthermore, some learners reported using energy drinks without any justified purpose, suggesting that the intake of energy drinks becomes a part of daily actions and common practice. In other words, this indicates that the high usage of energy drinks becomes more habitual regardless of specific needs.

### Strengths and Limitations

This study prompted the schools’ management to request the researcher to conduct awareness talks concerning energy drinks to the pupils. Thus, our results provide strong support for the necessity to address issues related to energy drink usage among pupils in the Mahikeng sub-district. The study was conducted as a cross-sectional, which means its findings should be viewed with caution, as this design demonstrates only associations and not causal relationships between variables. The study utilized self-administered questionnaires which rely on the honesty of the participants.

## 5. Conclusions

It is concluded that energy drink brands such as Dragons, Switch, and Red Bull are the most consumed energy drinks by pupils who participated in this study. It is also concluded that factors and reasons influencing energy drink usage among pupils include all-night parties, concentration, being awake, curiosity, energy levels, exams, sports, fatigue, and health. It is thus concluded that health education and promotion intervention programmes are required to educate pupils about the dangers of energy drink usage to prevent public health risks. In South Africa, studies about factors associated with energy drink usage among pupils are scarce. Further studies, including research on primary school learners, are necessary considering that a substantial number of learners were exposed to energy drinks at an early age. Qualitative investigations should be carried out to explore the causal relationship and perceptions of this demographic concerning energy drinks in-depth. It is necessary to investigate this phenomenon of interest more to gather the evidence needed for drafting or reviewing a policy document or legislation to regulate energy drinks, even if it starts at a school level.

## Figures and Tables

**Table 1:** The profile of schools.

Variable	Frequency	Percentage
School (n = 505)		
A	82	16.24
B	30	5.94
C	66	13.07
D	63	12.48
E	91	18.02
F	29	5.74
G	86	17.03
H	22	4.36
I	36	7.13
Quintile (n = 505)		
1	95	18.81
2	122	24.16
3	113	22.38
4	175	34.65

**Table 2:** Socio-demographics information of participants.

Variable	Frequency	Percentage (%)
**Age (n = 505)**		
13–15 years	148	29.31
16–18 years	232	45.94
Above 18 years	125	24.75
**Age (Mean = 16.86, SD = 2.20, Min = 13, Max = 24)**		
**Gender (n = 505)**		
Female	324	64.16
Male	181	35.84
**Race (n = 505)**		
Black	481	95.25
Coloured	24	4.75
**Grade (n = 505)**		
8	74	14.65
9	44	8.71
10	114	22.57
11	145	28.71
12	128	25.35
**Area (n = 505)**		
Rural	417	82.57
Urban	88	17.43
**Transport mode (n = 505)**		
Bus	53	10.50
Hitchhike	1	0.20
Parents’ car	48	9.50
Taxi	102	20.20
Walk	301	59.60
**Participation in Sports (n = 505)**		
Yes	153	30.30
No	352	69.70
**Sports Category (n = 153)**		
Athletics	22	14.38
Basketball	2	1.31
Board games	1	0.65
Boxing	1	0.65
Netball	60	39.22
Soccer	67	43.79
**Stay with (n = 505)**		
Alone	5	0.99
Both parents	175	34.65
Grandparents	63	12.48
Guardians	27	5.35
Older siblings	21	4.16
One parent	206	40.79
Other relatives	8	1.58
**Parent/Guardian Employment status (n = 505)**		
One working	224	44.36
Both working	121	23.96
Not working	160	31.68

**Table 3:** Reasons for consumption of energy drinks.

Reason for Consumption of Energy Drink	Frequency (%)
**Exam**	
Yes	54 (13.8%)
No	336 (86.2%)
**Alert**	
Yes	52 (13.3%)
No	338 (86.7%)
**Concentrate**	
Yes	79 (20.3%)
No	311 (79.7%)
**Energy**	
Yes	90 (23.1%)
No	300 (76.9%)
**Awake**	
Yes	168 (43.1%)
No	222 (56.9%)
**Curiosity**	
Yes	8 (2.1%)
No	382 (97.9%)
**Fatigue**	
Yes	27 (6.9%)
No	363 (93.1%)
**Healthy**	
Yes	9 (2.3%)
No	381 (97.7%)
**Sports Performance**	
Yes	34 (8.7%)
No	356 (91.3%)
**All night Party**	
Yes	12 (3.1%)
No	378 (96.9%)
**No reason**	
Yes	44 (11.3%)
No	346 (88.7%)

**Table 4:** Factors associated with the current consumption status of energy drinks.

Factor	Frequency (%)	Consume ED (%)	Do Not Consume ED (%)	Chi^2^	*p*-Value
		Grade		11.0577	0.026
**8**	68 (14.32%)	58 (14.87%)	10 (11.76%)		
**9**	44 (9.26%)	29 (7.44%)	15 (17.65%)		
**10**	107 (22.53%)	87 (22.31%)	20 (23.53%)		
**11**	135 (28.42%)	118 (30.26%)	17 (20.00%)		
**12**	121 (25.47%)	98 (27.06%)	23 (27.06%)		
		Participation in Sports			
**Yes**	148 (31.16%)	132 (33.85%)	16 (18.82%)	7.3428	0.007
**No**	327 (68.84%)	258 (66.15%)	69 (81.18%)		
		Knowledge score		11.8110	0.003
**Poor**	70 (14.74%)	67 (17.18%)	3 (3.53%)		
**Average**	125 (26.32%)	104 (26.67%)	21 (24.71%)		
**Good**	280 (58.95%)	219 (56.15%)	61 (71.76%)		

**Table 5:** Logistic regression model for current energy drink consumption status.

Factor	Coef.	Std. Err.	*p* > |z|	95% Conf. Interval	
**Grade**	−0.0928026	0.0923958	0.315	−0.273895	0.0882897
**Sports**	0.8001149	0.3033643	0.008	0.2055318	1.394698
**Knowledge score**	0.6244709	0.1999632	0.002	0.2325501	1.016392
**_cons**	−2.120171	1.016197	0.037	−4.111881	−0.1284621

**Table 6:** Factors associated with the frequency of energy drink consumption.

		Frequency of Consumption					
Factor	Frequency (%)	Everyday (%)	More than Once per Week but Not Every Day (%)	Once per Month (%)	Less Often than Once per Month (%)	Chi^2^	*p*-Value
			School Attended				
A	66 (16.92%)	1 (4.00%)	32 (19.28%)	25 (16.89%)	8 (15.69%)	40.2002	0.020
B	16 (4.10%)	1 (4.00%)	6 (3.61%)	8 (5.41%)	1 (1.96%)		
C	53 (13.59%)	6 (24.00%)	32 (19.28%)	10 (6.76%)	5 (9.80%)		
D	50 (12.82%)	4 (16.00%)	18 (10.84%)	17 (11.49%)	11 (21.57%)		
E	76 (19.49%)	7 (28.00%)	34 (20.48%)	24 (16.22%)	11 (21.57%)		
F	20 (5.13%)	3 (12.00%)	8 (4.82%)	8 (5.13%)	1 (1.96%)		
G	60 (15.38%)	2 (8.00%)	20 (12.05%)	33 (22.30%)	5 (9.80%)		
H	20 (5.13%)	0 (0.00%)	7 (4.22%)	11 (7.43%)	2 (3.92%)		
I	29 (7.44%)	1 (4.00%)	9 (5.42%)	12 (8.11%)	7 (13.73%)		
			Quintile of the school			19.7177	0.020
1	73 (18.72%)	9 (36.00%)	40 (24.10%)	18 (12.16%)	6 (11.76%)		
2	89 (22.82%)	3 (12.00%)	29 (17.47%)	45 (30.41%)	12 (23.53%)		
3	96 (24.62%)	7 (28.00%)	41 (24.70%)	35 (23.65%)	13 (25.49%)		
4	132 (33.85)	6 (24.00%)	56 (33.73%)	50 (33.78%)	20 (39.22%)		
			Age Category of learners				
13–15 years	106 (27.18%)	4 (16.00%)	41 (24.70%)	48 (32.43%)	13 (25.49%)	13.4123	0.037
16–18 years	184 (47.18%)	8 (32.00%)	81 (48.80%)	71 (47.97%)	24 (47.06%)		
Above 18 years	100 (25.64%)	13 (52.00%)	44 (26.51%)	29 (19.59%)	14 (27.45%)		
			Amount of energy drinks consumed in a day				
1	336 (86.15%)	18 (72.00%)	132 (79.52%)	138 (93.24%)	48 (94.12%)	35.7454	0.000
2	50 (12.82%)	5 (20.00%)	33 (19.88%)	9 (6.08%)	3 (5.88%)		
3	3 (0.77%)	1 (4.00%)	1 (0.60%)	1 (0.68%)	0 (0.00%)		
4 or more	1 (0.26%)	1 (4.00%)	0 (0.00%)	0 (0.00%)	0 (0.00%)		
			Effects attained after drinking an energy drink				
Always	76 (19.49%)	10 (40.00%)	38 (22.89%)	20 (13.51%)	8 (15.69%)	35.0745	0.000
Sometimes	224 (57.44%)	14 (56.00%)	101 (60.84%)	90 (60.81%)	19 (37.25%)		
Never	30 (7.69%)	0 (0.00%)	11 (6.63%)	11 (7.43%)	8 (15.69%)		
Do not know	60 (15.38%)	1 (4.00%)	16 (9.64%)	27 (18.24%)	16 (31.37%)		
			Reason for using energy drinks: No reason				
Yes	44 (11.28%)	1 (4.00%)	13 (7.83%)	18 (12.16%)	12 (23.53%)	11.0567	0.011
No	346 (88.72%)	24 (96.00%)	153 (92.17%)	130 (87.84%)	39 (76.47%)		
			Reason for using energy drinks: Exam				
Yes	54 (13.85%)	8 (32.00%)	23 (13.86%)	18 (12.16%)	5 (9.80%)	7.9572	0.047
No	336 (86.15%)	17 (68.00%)	143 (86.14%)	130 (87.84%)	46 (90.20%)		

**Table 7:** Logistic regression model for frequency of energy drink consumption.

Factor	Coef.	Std. Err.	*p* > |z|	95% Conf. Interval	
**Age Cat**	−0.7495682	0.3345011	0.025	−1.405178	−0.0939582
**Quintile**	0.1548623	0.1976006	0.433	−0.2324276	0.5421523
**School**	0.0470231	0.1081647	0.664	−0.1649758	0.2590219
**ED/Day**	−0.3144072	0.4627755	0.497	−1.22143	0.5926161
**Effect**	0.8025509	0.3534466	0.023	0.1098084	1.495293
**No reason**	−0.8960514	1.075065	0.405	−3.00314	1.211037
**Exam**	1.19548	0.495345	0.016	0.2246212	2.166338
**_cons**	2.54892	1.67316	0.128	−0.7304128	5.828252

**Table 8:** Factors associated with the reasons for the usage of energy drinks.

Factors	Frequency	Alert	All Night Party	Awake	Concentrate	Curiosity	Energy	Exam	Fatigue	Healthy	No Reason	Sports	Two or More Reasons	Chi²	*p*-Value
	School Attended													128.0027	0.003
**A**	66 (16.92%)	4 (13.33%)	0 (0.00%)	21 (21.88%)	6 (12.24%)	0 (0.00%)	3 (10.34%)	3 (10.00%)	2 (22.22%)	1 (50.00%)	2 (9.52%)	0 (0.00%)	24 (22.22%)		
**B**	16 (4.10%)	1 (3.33%)	0 (0.00%)	4 (4.17%)	2 (4.08%)	0 (0.00%)	2 (6.90%)	1 (3.33%)	0 (0.00%)	0 (0.00%)	0 (0.00%)	0 (0.00%)	6 (5.56%)		
**C**	53 (13.59%)	9 (30.00%)	1 (33.33%)	12 (12.50%)	12 (24.49%)	2 (40.00%)	5 (17.24%)	3 (10.00%)	4 (44.44%)	0 (0.00%)	0 (0.00%)	3 (37.50%)	2 (1.85%)		
**D**	50 (12.82%)	4 (13.33%)	1 (33.33%)	8 (8.33%)	8 (16.33%)	0 (0.00%)	1 (3.45%)	2 (6.67%)	0 (0.00%)	0 (0.00%)	5 (23.81%)	0 (0.00%)	21 (19.44%)		
**E**	76 (19.49%)	7 (23.33%)	1 (33.33%)	17 (17.71%)	10 (20.41%)	2 (40.00%)	5 (17.24%)	4 (13.33%)	2 (22.22%)	0 (0.00%)	4 (19.05%)	0 (0.00%)	24 (22.22%)		
**F**	20 (5.13%)	1 (3.33%)	0 (0.00%)	4 (4.17%)	0 (0.00%)	0 (0.00%)	2 (6.90%)	7 (23.33%)	0 (0.00%)	0 (0.00%)	0 (0.00%)	1 (12.50%)	5 (4.63%)		
**G**	60 (15.38%)	1 (3.33%)	0 (0.00%)	16 (16.67%)	8 (16.33%)	0 (0.00%)	4 (13.79%)	3 (10.00%)	0 (0.00%)	1 (50.00%)	7 (33.33%)	3 (37.50%)	17 (15.74%)		
**H**	20 (5.13%)	2 (6.67%)	0 (0.00%)	5 (5.21%)	1 (2.04%)	0 (0.00%)	4 (13.79%)	4 (13.33%)	0 (0.00%)	0 (0.00%)	1 (4.76%)	0 (0.00%)	3 (2.78%)		
**I**	29 (7.44%)	1 (3.33%)	0 (0.00%)	9 (9.38%)	2 (4.08%)	1 (20.00%)	3 (10.34%)	3 (10.00%)	1 (11.11%)	0 (0.00%)	2 (9.52%)	1 (12.50%)	6 (5.56%)		
	Quintile of the school													60.7848	0.002
**1**	73 (18.72%)	10 (33.33%)	1 (33.33%)	16 (16.67%)	12 (24.49%)	2 (40.00%)	7 (24.14%)	10 (33.33%)	4 (44.44%)	0 (0.00%)	0 (0.00%)	4 (50.00%)	7 (6.48%)		
**2**	89 (22.82%)	2 (6.67%)	0 (0.00%)	25 (26.04%)	10 (20.41%)	1 (20.00%)	7 (24.14%)	6 (20.00%)	1 (11.11%)	1 (50.00%)	9 (42.86%)	4 (50.00%)	23 (21.30%)		
**3**	96 (24.62%)	9 (30.00%)	1 (33.33%)	22 (22.92%)	11 (22.45%)	2 (40.00%)	9 (31.03%)	8 (26.67%)	2 (22.22%)	0 (0.00%)	5 (23.81%)	0 (0.00%)	27 (25.00%)		
**4**	132 (33.85%)	9 (30.00%)	1 (33.33%)	33 (34.38%)	16 (32.65%)	0 (0.00%)	6 (20.69%)	6 (20.00%)	2 (22.22%)	1 (50.00%)	7 (33.33%)	0 (0.00%)	51 (47.22%)		
	Amount of energy drinks consumed in a day													51.8232	0.020
**1**	336 (86.15%)	27 (90.00%)	3 (100.00%)	80 (83.33%)	46 (93.88%)	5 (100.00%)	28 (96.55%)	24 (80.00%)	5 (55.56%)	1 (50.00%)	21 (100.00%)	7 (87.50%)	89 (82.41%)		
**2**	50 (12.82%)	3 (10.00%)	0 (0.00%)	15 (15.62%)	3 (6.12%)	0 (0.00%)	1 (3.45%)	6 (20.00%)	3 (33.33%)	1 (50.00%)	0 (0.00%)	0 (0.00%)	18 (16.67%)		
**3**	3 (0.77%)	0 (0.00%)	0 (0.00%)	0 (0.00%)	0 (0.00%)	0 (0.00%)	0 (0.00%)	0 (0.00%)	1 (11.11%)	0 (0.00%)	0 (0.00%)	1 (12.50%)	1 (12.50%)		
**4 or more**	1 (0.26%)	0 (0.00%)	0 (0.00%)	1 (1.04%)	0 (0.00%)	0 (0.00%)	0 (0.00%)	0 (0.00%)	0 (0.00%)	0 (0.00%)	0 (0.00%)	0 (0.00%)	0 (0.00%)		
	Effects attained after drinking energy drinks													90.5044	0.000
**Always**	76 (19.49)	6 (20.00%)	1 (33.33%)	16 (16.67%)	11 (22.45%)	1 (20.00%)	7 (24.14%)	4 (13.33%)	1 (11.11%)	0 (0.00%)	1 (4.76%)	1 (12.50%)	27 (25.00%)		
**Sometimes**	224 (57.44%)	20 (66.67%)	1 (33.33%)	59 (64.46%)	29 (59.18%)	3 (60.00%)	16 (55.17%)	23 (76.67%)	8 (88.89%)	2 (100.00%)	1 (4.76%)	3 (37.50%)	59 (54.63%)		
**Never**	30 (7.69%)	1 (3.33%)	1 (33.33%)	10 (10.42%)	0 (0.00%)	0 (0.00%)	3 (10.34%)	0 (0.00%)	0 (0.00%)	0 (0.00%)	4 (19.05%)	2 (25.00%)	9 (8.33%)		
**Do not know**	60 (15.38%)	3 (10.00%)	0 (0.00%)	11 (11.46%)	9 (18.37%)	1 (20.00%)	3 (10.34%)	3 (10.00%)	0 (0.00%)	0 (0.00%)	15 (71.43%)	2 (25.00%)	13 (12.04%)		
	Brand of energy drink													175.2669	0.000
**Burn**	1 (0.26%)	0 (0.00%)	0 (0.00%)	1 (1.04%)	0 (0.00%)	0 (0.00%)	0 (0.00%)	0 (0.00%)	0 (0.00%)	0 (0.00%)	0 (0.00%)	0 (0.00%)	0 (0.00%)		
**Dragon**	149 (38.21%)	11 (36.67%)	1 (33.33%)	40 (41.67%)	22 (44.90%)	1 (20.00%)	10 (34.48%)	16 (53.33%)	2 (22.22%)	1 (50.00%)	8 (38.10%)	1 (12.50%)	36 (33.33%)		
**Mofaya**	18 (4.62%)	1 (3.33%)	0 (0.00%)	5 (5.21%)	4 (8.16%)	0 (0.00%)	0 (0.00%)	0 (0.00%)	0 (0.00%)	0 (0.00%)	0 (0.00%)	4 (50.00%)	4 (3.70%)		
**Monster**	29 (7.44%)	3 (10.00%)	0 (0.00%)	6 (6.25%)	1 (2.04%)	1 (20.00%)	2 (6.90%)	3 (10.00%)	0 (0.00%)	0 (0.00%)	1 (4.76%)	0 (0.00%)	12 (11.11%)		
**Power play**	18 (4.62%)	0 (0.00%)	0 (0.00%)	6 (6.25%)	1 (2.04%)	0 (0.00%)	1 (3.45%)	1 (3.33%)	1 (11.11%)	0 (0.00%)	0 (0.00%)	0 (0.00%)	8 (7.41%)		
**Predator**	1 (0.26%)	0 (0.00%)	0 (0.00%)	0 (0.00%)	0 (0.00%)	0 (0.00%)	0 (0.00%)	0 (0.00%)	0 (0.00%)	0 (0.00%)	1 (4.76%)	0 (0.00%)	0 (0.00%)		
**Prime**	3 (0.77%)	0 (0.00%)	0 (0.00%)	0 (0.00%)	0 (0.00%)	0 (0.00%)	0 (0.00%)	0 (0.00%)	0 (0.00%)	1 (50.00%)	0 (0.00%)	0 (0.00%)	2 (1.85%)		
**Reboost**	1 (0.26%)	0 (0.00%)	0 (0.00%)	0 (0.00%)	0 (0.00%)	0 (0.00%)	0 (0.00%)	0 (0.00%)	0 (0.00%)	0 (0.00%)	0 (0.00%)	0 (0.00%)	1 (0.93%)		
**Red bull**	57 (14.62%)	7 (23.33%)	0 (0.00%)	5 (5.21%)	12 (24.49%)	1 (20.00%)	6 (20.69%)	5 (16.67%)	1 (11.11%)	0 (0.00%)	3 (14.29%)	1 (12.50%	16 (14.81%)		
**Switch**	113 (28.97%)	8 (26.67%)	2 (66.67%)	33 (34.38%)	9 (18.37%)	2 (40.00%)	10 (34.48%)	5 (16.67%)	5 (55.56%)	0 (0.00%)	8 (38.10%)	2 (25.00%)	29 (26.85%)		

**Table 9:** Logistic regression model for reasons for energy drink consumption.

Reasons for Consumption	Coefficient	Std. Err.	*p* > z	95% Conf. Interval	
**Quintile**	0.2482413	0.170538	0.145	−0.086007	0.5824896
**School**	0.1434969	0.0903775	0.112	−0.0336397	0.3206334
**ED/Day**	0.398107	0.5729218	0.487	−0.7247991	10.521013
**Effect**	0.1390652	0.2305383	0.546	−0.3127816	0.5909121
**Brand**	−0.0291813	0.0541465	0.590	−0.1353065	0.0769439
**_cons**	0.8801455	0.9197154	0.339	−0.9224636	20.682755

## Data Availability

Data will be made available on request. The data remains the university property since we promised the participants after data collection, we will publish the findings of the study and data sets and their personal data will remain at the university storage.

## Supplementary Materials

The following supporting information can be downloaded at: www.mdpi.com/article/10.3390/nu17050770/s1, Supplementary S1: Information leaflet; Supplementary S2: Informed consent; Supplementary S3: Assent forms; Supplementary S4: Socio-demographics questionnaire; Supplementary S5: Data collection questionnaire.
