# Supplementary material for: Factors Influencing Energy Drink Usage Amongst Pupils in the Mahikeng Sub-District, Northwest
Source: Nutrients. 2025 Feb 21;17(5):770. doi: 10.3390/nu17050770 (PMC11901862; doi:10.3390/nu17050770)
Supplement: Supplementary file 1 [file nutrients-17-00770-s001.zip › Supplementary S1.pdf]

Information leaflet

My name is Karabo Dina Thini, a student at Sefako Makgatho Healthcare Sciences University situated in Ga-Rankuwa in Gauteng Province. I am conducting a research study that aims to determine the usage of energy drinks among high school learners. The results of this study will provide information that can assist us to understand how much of energy drinks learners are consuming and why.

Your child is a learner at ____________________________________ School and the study requires that I collect information from him/her in this regard. The study will be conducted at school and information will be collected in a form of a questionnaire where the learner will participate by answering questions on paper about the use of energy drinks. The study is voluntary and confidential with no monetary benefit attached to it. It is purely for educational purposes. The study will be anonymous, and no learner and school details (names) will be reported in the study.

We have obtained permission from the District Office of Education, as well as the management of the school, and hereby request that you allow your child to participate in the study.

Attached is a form that we ask you to fill out and sign, as an indication that you give permission for your child to participate in the study.

Thank you.
