# Supplementary material for: Factors Influencing Energy Drink Usage Amongst Pupils in the Mahikeng Sub-District, Northwest
Source: Nutrients. 2025 Feb 21;17(5):770. doi: 10.3390/nu17050770 (PMC11901862; doi:10.3390/nu17050770)
Supplement: Supplementary file 1 [file nutrients-17-00770-s001.zip › Supplementary S2.pdf]

SEFAKO MAKGATHO
HEALTH SCIENCES UNIVERSITY

# SEFAKO MAKGATHO HEALTH SCIENCES UNIVERSITY ENGLISH CONSENT FORM

**Your child is hereby kindly invited to participate in a Research Project**

Name of Study

**The usage of energy drinks among high school learners in Mahikeng sub-district of Northwest Province.**
................................................................................................................................................................

I have read the information on the aims and objectives of the proposed study and was provided the opportunity to ask questions and given adequate time to rethink the issue. The aim and objectives of the study are sufficiently clear to me. I have not been pressurized for my child to participate in any way.

I am aware that this material may be used in scientific publications which will be electronically available throughout the world. I consent to this provided that my child's name is not revealed.

I understand that participation in this Study is completely voluntary and that my child may withdraw from it at any time and without supplying reasons. This will have no influence on the regular treatment and the care that my child receives at his/her school.

I know that this Study has been approved by the Sefako Makgatho University Research Ethics Committee (SMUREC), the Department of Basic Education in Northwest Province Ngaka Modiri Molema District, and the School Management. I am fully aware that the results of this study will be used for scientific purposes and may be published. I agree to this, provided my child's privacy is guaranteed.

I hereby give consent for my child ________________________________________ to participate in this Study.

| | |
|---|---|
| ............................................................ | ........................................................ |
| Name of parent/guardian | Signature of parent or guardian |

| | | |
|---|---|---|
| ................................ | .................................... | ................................................ |
| Place | Date | Witness |

---

**Statement by the Researcher**

I provided written information regarding this Study
I agree to answer any future questions concerning the Study as best as I am able.
I will adhere to the approved protocol.

| | | | |
|---|---|---|---|
| Karabo Thini | .................................... | ...................... | …………………. |
| Name of Researcher | Signature | Date | Place |
