# Supplementary material for: Factors Influencing Energy Drink Usage Amongst Pupils in the Mahikeng Sub-District, Northwest
Source: Nutrients. 2025 Feb 21;17(5):770. doi: 10.3390/nu17050770 (PMC11901862; doi:10.3390/nu17050770)
Supplement: Supplementary file 1 [file nutrients-17-00770-s001.zip › Supplementary S3.pdf]

SEFAKO MAKGATHO HEALTH SCIENCES UNIVERSITY

ASSENT FORM FOR MINORS

Project Title: The usage of energy drinks among high school learners in Mahikeng sub-district of Northwest Province.

Researcher: Karabo Thini

I am conducting a study about the usage of energy drinks among high school learners. A research study is a way to learn more about people. If you decide that you want to be part of this study, you will be asked to sign a form voluntarily.

There are some things about this study you should know. I will ask you questions and I humbly ask you to be honest. Your answers will benefit this study and may be used for publications in conferences and worldwide. There is no monetary benefit, no harm will be imposed as you are not exposed to any dangers of investigation.

When we are finished with this study, we will write a report about what was learned. This report will not include your name or that you were in the study. You do not have to be in this study if you do not want to be. If you decide to stop after we begin, that's okay too. Your parents know about the study too. If you decide you want to be in this study, please sign your name.

I, ___________________________________, want to participate in this research study.

______________________________________ ______________________________

(Participant's signature) (Date)

Witness_______________________________ Date_______________________
