# Supplementary material for: Factors Influencing Energy Drink Usage Amongst Pupils in the Mahikeng Sub-District, Northwest
Source: Nutrients. 2025 Feb 21;17(5):770. doi: 10.3390/nu17050770 (PMC11901862; doi:10.3390/nu17050770)
Supplement: Supplementary file 1 [file nutrients-17-00770-s001.zip › Supplementary S4.pdf]

SEFAKO MAKGATHO HEALTH SCIENCES UNIVERSITY

**Sociodemographic Questionnaire**						
**Please tick the correct answer**						
1.	What is the name of your school?					
2.	How old are you?					
3.	What is your gender?	Male ☐			Female ☐	
4.	What is your race?	Asian ☐	Black ☐	Coloured ☐	White ☐	Other (specify) ☐ ______
5.	What grade are you doing?	Grade 8 ☐	Grade 9 ☐	Grade 10 ☐	Grade 11 ☐	Grade 12 ☐
6.	How do you get to school?	Walk ☐	Taxi ☐	Bus ☐	Parent's car ☐	Other (specify) ☐ ______
7.	Are you playing any sports in school?	Yes ☐			No ☐	
8.	What sport are you playing?	Soccer ☐	Netball ☐	Athletics ☐	Others (specify) ☐ ______	
9.	Where are you staying?	Urban ☐			Rural ☐	
10.	Who do you stay with at home?	Both Parents ☐	One Parent ☐	Grand Parents ☐	Older Siblings ☐	
		Guardians ☐	Alone ☐	Other relatives ☐	Other (specify) ☐ ______	
11.	What is the employment status of your parents/guardians?	Both working ☐	One working ☐	Both not working ☐	Other (specify) ☐ ______	
