# Supplementary material for: Factors Influencing Energy Drink Usage Amongst Pupils in the Mahikeng Sub-District, Northwest
Source: Nutrients. 2025 Feb 21;17(5):770. doi: 10.3390/nu17050770 (PMC11901862; doi:10.3390/nu17050770)
Supplement: Supplementary file 1 [file nutrients-17-00770-s001.zip › Supplementary S5.pdf]

SEFAKO MAKGATHO
HEALTH SCIENCES UNIVERSITY

Data Collection Questionnaire

SECTION A: USE

Please Click the correct answer

1.	Have you ever consumed an energy drink?	Yes ☐		No ☐ If "No" move to Section C	
2.	How old were you when you had your first energy drink?				
3.	How were you first introduced to energy drinks?	Advertisement e.g., TV, radio ☐	By a friend ☐	By family member ☐	By myself ☐
		At a social gathering e.g., party ☐	Free sample e.g., promotion ☐	Social media e.g., Facebook, Tiktok, Twitter ☐	Other (specify) ____________ ☐
4.	Are you currently consuming energy drinks?	Yes ☐		No ☐	
5.	**If you answered "No" to question 4, answer this question and move to Section C** Why did you stop consuming energy drinks?	No reason (free will) ☐	Made me sick ☐	Advise from someone e.g., parents, friend ☐	Other (specify) ____________ ____________ ☐
6.	How often do you consume energy drinks?	Everyday ☐	More than once per week **but not every day** ☐	Once per month ☐	Less often **than once per month** (specify) ☐ ____________ ____________
7.	During the past 7 days, how many times did you drink an energy drink?	I did not drink energy drinks in the past 7 days ☐	1-3 times in a week ☐	4-6 times in a week ☐	Everyday ☐
8.	How many energy drinks do you typically have in one day?	1 can ☐	2 cans ☐	3 cans ☐	4 or more cans ☐
9.	What type/brand of energy drink do you mainly consume?	Red bull ☐	Monster ☐	Dragon ☐	Mofaya ☐
		Power play ☐	Switch ☐	Prime ☐	Other (specify) ☐ ____________

**SECTION B: REASONS**					
10.	What is your reason for selecting your preferred brand?	Taste ☐	Price (tick the applicable price preference) Cheaper brand ☐ OR Expensive brand ☐	Strong effect ☐ Specify the effect: ____________ ____________ ____________	Other (specify) ☐ ____________ ____________
11.	Why do you usually drink energy drinks? **(Select all that is applicable to you)**	It helps me to perform better in the exam/tests ☐	To be more alert ☐	To help me concentrate during study ☐	To help me stay awake ☐
		It gives me long-lasting energy all day ☐	Out of curiosity ☐	To reduce fatigue ☐	It makes me feel healthy ☐
		To perform better in sports ☐	To enjoy an all-night party ☐	I drink it without any particular reason ☐	Other (specify) ☐ ____________ ____________
12.	Your desired effect upon drinking the energy drink selected above are met/attained.	Always ☐	Sometimes ☐	Never ☐	Do not know ☐
**SECTION C: KNOWLEDGE**					
13.	Energy drinks are the same as soft drinks such as fanta, coca-cola, sprite, etc.	True ☐		False ☐	
14.	Energy drinks are safe to consume.	True ☐		False ☐	
15.	Energy drinks can cause serious health problems.	True ☐		False ☐	
16.	Energy drinks can be addictive.	True ☐		False ☐	
17.	Energy drinks can make you gain weight.	True ☐		False ☐	
**End of Questions**<br>**Thank you for taking the time to complete the questionnaire**					
